# Animal Physics
*Animal Physics; or the Body and Its Functions Familiarly Explained. By Dionysius Lardner, D.C.L. London: Walton and Maberly. 1857.


**Published:** 1857-07

**Authors:** 


### ANIMAL PHYSICS.*

Dr. Lardner's new volume on Animal Physics is a work long waited for and wanted. In a book of 705 pages, profusely and admirably illustrated with drawings, the public are presented with a masterly summary of the laws which govern life. Dr. Lardner's style of writing combines the pure scientific, with the equally pure common-sense trait of appealing to all intellects. The Animal Physics is a work which the professional and scientific man will read and study with immense pleasure; he will think it a learned and classical book. The mechanic too, visiting his reading room, will read it, and think it easy and popular. The book does not admit of condensation or analysis, since it is a pure recital of scientific facts. All we can say of it is, that it is the best general review in the English language of the physics of the animal world, and so leave it favourably recommended to readers.

### TORQUAY, A RESORT FOR CONSUMPTIVES.†

In this work one of our most accomplished provincial phy-

* Animal Physics; or the Body and Its Functions Familiarly Explained. By DIONYSIUS LARDNER, D.C.L. London: Walton and Maberly. 1857.

